# Proteasome Inhibition Is Partially Effective in Attenuating Pre-Existing Immunity against Recombinant Adeno-Associated Viral Vectors

**DOI:** 10.1371/journal.pone.0034684

**Published:** 2012-04-13

**Authors:** Jozsef Karman, Nathan K. Gumlaw, Jinhua Zhang, Ji-Lei Jiang, Seng H. Cheng, Yunxiang Zhu

**Affiliations:** Genetic Disease Science, Genzyme Corporation, Framingham, Massachusetts, United States of America; Leiden University Medical Center, The Netherlands

## Abstract

Pre-existing immunity against adeno-associated virus (AAV) remains a major challenge facing the clinical use of systemic administration of recombinant AAV vectors for the treatment of genetic and acquired diseases using gene therapy. In this study, we evaluated the potential of bortezomib (marketed under trade name Velcade) to abrogate a pre-existing immunity to AAV in mice, thereby allowing subsequent transduction by a recombinant AAV vector of the same serotype. We demonstrate that bortezomib efficiently reduces AAV-specific IgG titres and moderates the cytotoxic T cell response in mice that have a pre-existing immunity to AAV2/8. Significant depletion of AAV2/8-specific IgG-producing plasma cells in secondary lymphoid organs and bone marrow was observed. However, this inhibition of the immune response by bortezomib was insufficient to allow subsequent re-infection with a recombinant AAV vector of a similar serotype. We show that this shortcoming is probably due to the combination of residual antibody levels and the inability of bortezomib to completely deplete the memory B cells that are re-activated in response to a repeated infection with a recombinant AAV vector. Taken together, the results of this study argue for the use of immunosuppressive therapies that target both plasma and memory B cells for the efficient elimination of pre-existing immunity against AAV2/8 vectors.

## Introduction

Recombinant adeno-associated viral (AAV) vectors have been extensively studied as delivery systems for gene therapy treatment in a variety of disease indications. This vector platform is being used in several clinical trials for the treatment of a number of genetic diseases, including haemophilia B, α-1-antitrypsin deficiency, Duchenne muscular dystrophy, Leber’s congenital amaurosis, leukodystrophies, age-related macular degeneration and Parkinson’s disease [1]–[5]. This progress to clinical trials has stemmed from pre-clinical studies showing that recombinant AAV vectors transduce a variety of cell types, facilitate high-level and long-term gene expression and are relatively innocuous [1]–[5].

Although certain characteristics of AAV make it a promising gene transfer vector, several challenges limit its clinical use. Foremost, there is a prevalence of pre-existing immunity to the viral capsids in the general population [6]. This pre-existing immunity to AAV may be due to endemic infection by the virus early in childhood [6]. To alleviate this pre-existing immunity, capsid proteins have been modified to exhibit a lower immunogenicity profile, and immunosuppressive agents have been deployed to dampen the immune response [7], [8]. The immunosuppressive agents that have been evaluated are primarily agents that inhibit T cell activation/survival and inhibit antigen presentation on major histocompatibility (MHC) molecules to T cells. The agent bortezomib (Velcade) [9] has been used successfully in several pre-clinical studies. Bortezomib, a specific inhibitor of the 26 S proteasome, has been approved as a treatment for patients with multiple myeloma [9]. In pre-clinical studies, when bortezomib was administered simultaneously with a recombinant AAV vector, it reportedly increased the efficacy of gene delivery [10]–[12]. In these studies, bortezomib inhibited cytotoxic CD8^+^ T cell responses through its activity against the 26 S proteasome system, which prevented antigen presentation on MHC class I molecules to CD8^+^ T cells [10].

Recently, bortezomib has been shown to exhibit additional inhibitory effects on the immune system. In a pre-clinical animal model of systemic lupus erythematosus (SLE), bortezomib was highly efficient at depleting antibody-producing cells in secondary lymphoid organs and bone marrow, thereby limiting the progression of the disease [13]. It was suggested that this effect was due to the susceptibility of terminally differentiated B cells (which produce large amounts of secreted antibody) to proteasome inhibition. This increased susceptibility of antibody-producing cells to bortezomib was due to their dependence on the unfolded protein response that eliminates misfolded proteins, which was blocked by proteasome inhibition [13]–[15]. Based on these observations, we argue that this property of bortezomib might be advantageous in promoting the efficacy of AAV-mediated gene therapy. Similar to the antibody-producing cells in SLE, bortezomib might act to deplete antibody-producing cells that constitute the pre-existing anti-AAV humoral immunity, thereby enabling increased gene transduction activity. In this study, we demonstrate that bortezomib lowers pre-existing anti-AAV antibody levels in mice by reducing the number of plasma cells in secondary lymphoid organs and bone marrow. However, this reduction is insufficient to support a subsequent infection by a recombinant AAV2/8 vector. This inability to support a subsequent infection is probably due to the combination of residual antibody levels after bortezomib treatment and the inability of bortezomib to influence memory B cell populations that are re-activated in response to a secondary challenge by the recombinant AAV2/8 serotype vector.

## Materials and Methods

### Animals

Ethics Statement: Procedures involving mice were reviewed and approved by Genzyme Corporation’s Institutional Animal Care and Use Committee (protocol number 10-0224-01) following guidelines established by the Association for Assessment of Accreditation of Laboratory Animal Care. The review board specifically approved all the studies (identification numbers 10-00605 and 10-01005) reported in this manuscript. Wild-type male C57BL/6 mice between the ages of 6 and 10 weeks were obtained from Jackson Laboratories (Bar Harbor, ME). All animal experiments were approved by Genzyme Corporation’s Institutional Animal Care and Use Committee. At least 7 animals per group were used in each experiment.

### Recombinant AAV Vectors

Recombinant AAV2/8 serotype vectors were generated and titred at Genzyme Corporation, as described previously [16], [17]. Two AAV2/8 vectors were used in the studies: (1) AAV2/8-EV (an empty vector) harbouring a human cytomegalovirus promoter but no transgene and (2) AAV2/8-DC190-alphaGal designed to express the lysosomal enzyme α-galactosidase A under the transcriptional control of a liver-specific promoter (DC190) [16], [17]. For all studies, the mice were injected intravenously with 5×10^11^ viral particles via the tail vein.

### Bortezomib Treatment

Male mice were infected with AAV2/8-EV as described above. At four weeks post-administration of the virus, the animals were treated with various doses of bortezomib (Selleck Chemicals, Houston, TX) administered by intravenous injection twice weekly for 20 weeks. At the end of the 20-week period (24 weeks post-infection with AAV2/8-EV), the mice were administered 5×10^11^ viral particles of AAV2/8-DC190-alphaGal three days after the final treatment with bortezomib. Serum levels of α-galactosidase A were monitored starting at one and two weeks post-challenge. At four weeks post-challenge, the mice were sacrificed, and immune responses were evaluated, as described below.

### Flow Cytometry and Measurement of Cytokine Levels

Fluorescently labelled antibodies were obtained from either eBioscience (San Diego, CA) or BD Biosciences (San Jose, CA). Flow cytometry was performed by staining 10^6^ cells that were suspended in PBS containing 1% bovine serum albumin. Fc receptors were blocked prior to staining using unlabelled blocking antibody. Cells were stained with a combination of fluorescently labelled anti-CD3, CD4, CD8, CD44, CD27, CD19, CD38, and CD138 antibodies for 20 minutes at 4°C, thoroughly washed and analysed using an LSRII cytometer (BD Biosciences, San Jose, CA) and FlowJo version 7.6.4 software (Treestar, Eugene, OR).

Mononuclear cells prepared from spleens were reactivated by being exposed to various amounts of recombinant AAV2/8-EV vectors (10, 1, and 0.1 µg/ml capsid protein, respectively) *in vitro* in Dulbecco’s modified Eagle’s medium with 10% fetal bovine serum. In the figures, results using 10 µg/ml capsid protein are shown. At three days post-activation, the cells were stained as described above, the supernatants were collected and the levels of interleukin-2 and interferon-γ were measured using an enzyme-linked immunosorbent assay according to the manufacturer’s instructions (eBioscience, San Diego, CA).

### Measurement of AAV2/8-specific IgG, Total IgG and α-galactosidase A Levels

Levels of AAV2/8-specific IgG were measured by coating high-binding enzyme immunoassay plates with AAV2/8-EV. Serum samples were titrated onto the coated plates and developed with horseradish peroxidase-conjugated goat anti-mouse IgG (Sigma-Aldrich, St. Louis, MO). Titres were determined as the lowest dilution of serum at which the OD was equal to or greater than 0.1. The levels of total IgG in serum and culture supernatants were measured by coating high-binding enzyme immunoassay plates with unlabelled rabbit anti-mouse IgG (Novus Biologicals, Littleton, CO), and the plates were developed using horseradish peroxidase-conjugated donkey anti-mouse IgG (Novus Biologicals, Littleton, CO). Serum α-galactosidase A levels were measured as described previously [17]. For the immunohistochemical studies, livers were harvested, sectioned and fixed in 4% paraformaldehyde. Paraffin-embedded sections (5 µm) were stained with rabbit anti-alpha galactosidase A antibody [16] followed by HRP-labelled anti-rabbit IgG (Sigma-Aldrich, St. Louis, MO).

### Statistical Analysis

Student’s *t*-test was used to compare groups of data. A *p* value of less than 0.05 was considered significant. Multiple groups of data were compared using one-way ANOVA.

## Results

### Bortezomib is Effective in Reducing Anti-recombinant AAV2/8 Vector Antibody Levels in Mice

**Figure 1:**
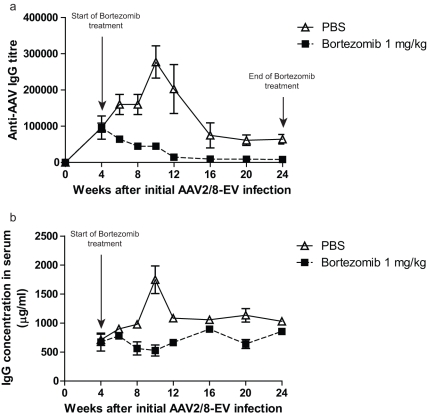
Bortezomib treatment significantly reduces anti-AAV IgG titre. a. Mice were infected with AAV2/8-EV and treated with bortezomib, as described in the Materials and Methods section. Sera were collected at the indicated times, and the levels of anti-AAV IgG were determined by titration. **b.** A subset of the same sera samples was assayed for the total amount of IgG using ELISA. Representative data from two separate experiments with similar outcomes are shown.

Bortezomib is effective in reducing the levels of antibody-producing cells as illustrated by its ability to lower autoantibody titres in a murine model of systemic lupus erythematosus [13]. Consequently, we tested whether a bortezomib-mediated reduction of antibody-producing cells would lead to a similar abatement of humoral immunity to recombinant AAV vectors. Mice were injected systemically with an empty AAV2/8 pseudotyped vector (containing no transgene) [16], [18] and were treated (by intravenous injection) with various amounts of bortezomib four weeks later. This timepoint was chosen as antibody levels steadily rise and immunological memory is formed in the four weeks post initial infection. Indeed, during the intervening 4-week period, a significant increase in anti-AAV2/8 antibodies was observed (Figure 1). Mice that were administered 1 mg/kg bortezomib did not exhibit an increase in antibody titre, and importantly, antibody levels were reduced to a steady state following an additional 8 weeks of treatment (Figure 1a). An 8- to 10-fold reduction in anti-AAV IgG levels was noted in the treated animals; however, these animals retained significant levels of antibodies. The observed reduction in AAV-specific IgG levels was correlated with a lower level of total IgG in the sera, suggesting that bortezomib inhibited all IgG antibody-producing cells (Figure 1b). The administration of lower doses (0.2 and 0.04 mg/kg) of bortezomib did not alter the profile of anti-AAV titres from that of the vehicle-treated controls (data not shown). However, toxicity was observed in ∼15% (1/7) of the animals that were treated with 1 mg/kg bortezomib, indicating that the drug displays a narrow therapeutic window in mice as a strategy to lower pre-existing anti-AAV immunity.

### Bortezomib Reduces the Number of CD138^+^ Plasma Cells but not Other B or T Cell Populations

**Figure 2:**
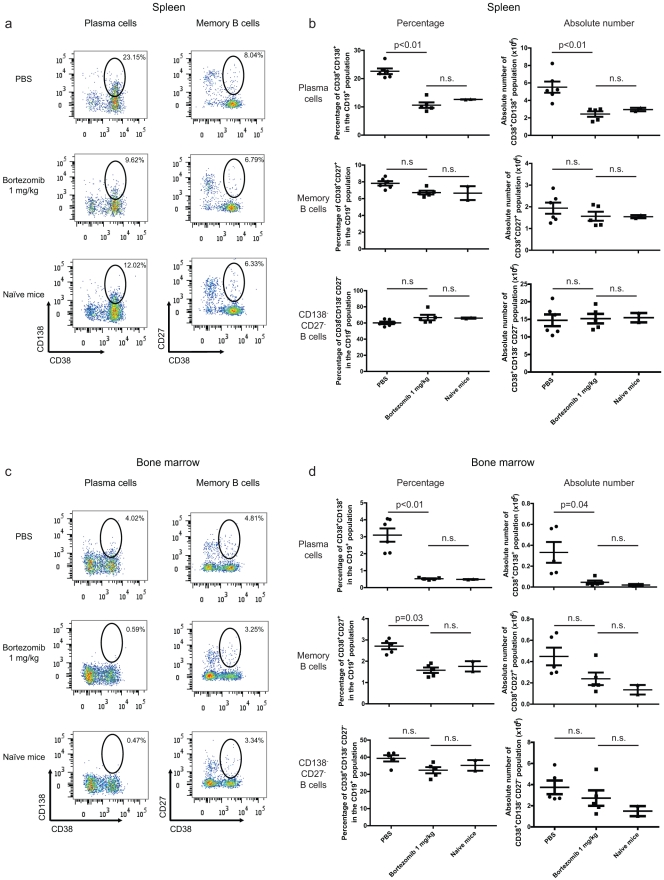
Bortezomib treatment reduces plasma cell levels in spleen and bone marrow. a. Mononuclear cells were prepared from spleen and analysed for the B cell markers CD19, CD38, CD138, and CD27 using flow cytometry. Plots depict CD27 and CD138 expression gated on the CD19^+^ population. **b.** A summary of the data from **a** is shown in a quantitative format. **c.** Mononuclear cells that were isolated from bone marrow were analysed for the B cell markers CD19, CD38, CD138, and CD27. Plots depict CD27 and CD138 expression gated on the CD19^+^ population. **d.** A summary of the data from **c** is shown in a quantitative format. The figures show representative data from two independent experiments with similar outcomes.

To determine the basis of the bortezomib-mediated reduction in anti-AAV IgG levels, B and T cell populations were analysed following termination of the *in vivo* phase of the studies. B cells are capable of secreting IgG at several developmental stages of their life cycle and require help from T cells for isotype switching [19]. To elucidate the specific juncture at which bortezomib may function, the percentage of various B and T cell populations in the spleen and bone marrow (terminally differentiated plasma cells home to niches in the bone marrow for long term survival [20]) of the mice was evaluated. Splenic and bone-marrow cell populations were incubated with AAV for three days *in vitro* to enrich for CD138^+^ cells. We elected using this method due to the fact that CD138^+^ cells constitute a very rare population in bone marrow that is close to level of detection by flow cytometry. Bortezomib-treated animals exhibited a significant decrease in the percentage and absolute numbers of CD138^+^ plasma cells in the spleen and bone marrow (Figure 2a, left panel and Figure 2b, top row; Figure 2c, left panel and Figure 2d, top row). Interestingly, no other B cell population (e.g., splenic CD27^+^ memory B cells and CD19^+^CD27^−^CD138^−^ non-memory/non-plasma cell B cells) was significantly reduced (Figure 2a, right panel and Figure 2b, middle and bottom rows; Figure 2c, right panel and Figure 2d, middle and bottom rows). In the bone marrow, a small but statistically significant decrease was detected in the percentage of CD27^+^ B cells, but there was no decrease in the absolute number of CD27^+^ B cells (Figure 2c and 2d, middle row). These results are congruent with studies by Neubert et al. [13] that demonstrated that bortezomib treatment has little or no effect on B cell populations in mice other than that on plasma cells.

**Figure 3:**
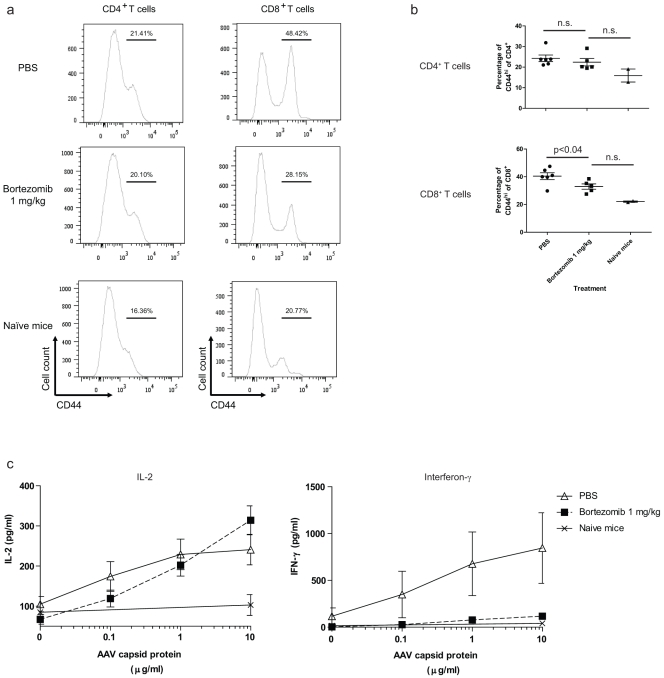
Bortezomib treatment differentially affects T cells. **a.** Mononuclear cells were prepared from spleens and analysed for the T cell markers CD4, CD8, and CD44 using flow cytometry. Plots depict CD44 expression gated on the CD4^+^ (left panel) and CD8^+^ (right panel) populations. **b.** A summary of the data from **a** is shown in a quantitative format. **c.** Mononuclear cells isolated from spleen were reactivated with AAV2/8-EV for 72 h *in vitro*. Supernatants were collected and assayed for IL-2 (left panel) or interferon-γ (right panel) levels using ELISA. The figures show representative data from two studies with similar outcomes.

T cell responses were evaluated by staining with anti-CD4, anti-CD8 and anti-CD44 (to assess T-cell activation status) antibodies. The response to *in vitro* reactivation was measured by assessing cytokine production. Direct *ex vivo* staining of splenic and bone marrow lymphocytes demonstrated differential effects on helper and cytotoxic T cells, respectively (Figure 3). The percentage or absolute number of CD4^+^ helper T cells did not change (data not shown). The activation status of the CD4^+^ T cell population, as assessed by CD44 expression, also remained unchanged (Figure 3a, left panel and Figure 3b, top panel). Compared to the PBS-treated controls, the percentage and absolute number of CD8^+^ cytotoxic T cells remained unchanged in the bortezomib-treated animals (data not shown). However, there was a significant reduction in the percentage of the CD44^high^ effector/memory CD8^+^ T cell population (Figure 3a, right panel and Figure 3b, bottom panel). This finding is consistent with previous reports that bortezomib has an inhibitory effect on cytotoxic T cell responses, presumably because antigen presentation to CD8^+^ T cells requires protein degradation by the 26 S proteasome [21], [22].

T cells from bortezomib- and PBS-treated mice produced similar levels of interleukin-2 following reactivation by AAV (Figure 3c, left panel). However, there was a significant decrease in the levels of the signature antiviral cytokine interferon-γ in the bortezomib-treated mice compared to the PBS-treated controls (Figure 3c, right panel). This result was probably due to the decrease in the number and percentage of the CD44^high^ fraction of the CD8^+^ T cell population, a major producer of this cytokine. These data suggest that bortezomib inhibits the immune response to AAV at the level of both plasma and CD8^+^ T cells. However, the effect of bortezomib on B cells is restricted to the terminally differentiated CD138^+^ plasma cell population; bortezomib does not affect other B cell subpopulations.

### Bortezomib-mediated Reduction of Pre-existing Immunity to AAV is Ineffective in Negating a Humoral Immune Response to a Subsequent Infection by a Recombinant AAV Vector

**Figure 4:**
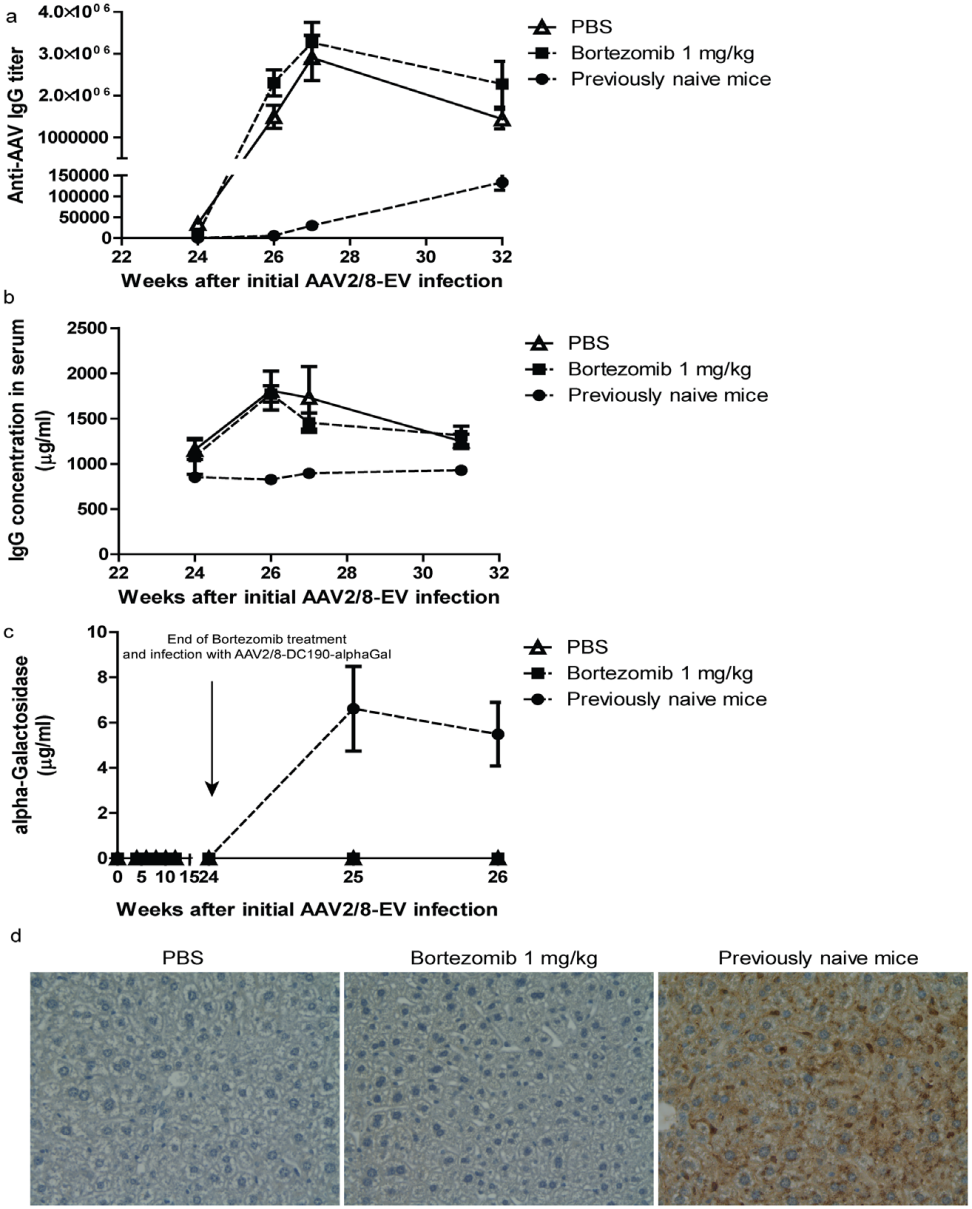
Bortezomib-mediated reduction in anti-AAV IgG titres is insufficient to facilitate reinfection with AAV2/8-DC190-alphaGal. **a.** At twenty-four weeks following the administration of AAV2/8-EV (including 20 weeks of bortezomib treatment between weeks 4 and 24), mice were re-infected with 5×10^11^ AAV2/8-DC190-alphaGal/mouse vector particles. Sera were collected at the indicated times, and the titres of anti-AAV IgG were determined. **b.** A subset of the sera samples was assayed for the total amount of IgG using ELISA. **c.** The level of α-galactosidase A in serum samples from mice infected with AAV2/8-DC190-alphaGal. Expression of α-galactosidase A was observed in mice that had not been previously infected with AAV2/8-EV. **d.** Immunohistochemistry was performed on liver sections from mice that were administered AAV2/8-DC190-alphaGal. The figures show representative data from two studies with similar outcomes.

A potential application for bortezomib is the depletion of pre-existing anti-AAV antibodies such that the host is conducive to a subsequent infection by a recombinant AAV vector of the same serotype. To test this theory, mice were first administered an empty viral vector (AAV2/8-EV) and were subsequently subjected to treatment with bortezomib 4 weeks later (following the appearance of anti-AAV antibodies) for 20 weeks. At the end of this 20-week period, the mice were challenged systemically with a recombinant AAV2/8 vector encoding α-galactosidase A (AAV2/8-DC190-alphaGal). At week 24 post infection, serum anti-AAV IgG titers were 1∶64,000±13,249 and 1∶8,000±1852 for the PBS- and bortezomib-treated groups, respectively (Figure 1a and 4a). A rapid and robust increase in anti-AAV-specific IgGs was observed in both bortezomib- and PBS-treated (control) animals following administration of AAV2/8-DC190-alphaGal (Figure 4a). The levels of IgG peaked between 2 and 3 weeks post-infection and remained undiminished at 8 weeks (Figure 4a). Previously naïve mice infected with the AAV2/8-DC190-alphaGal vector showed an increase in anti-AAV IgG levels with significantly slower kinetics as compared to animals previously exposed to AAV2/8-EV, indicating a primary immune response in the previously naïve animals as compared to a memory response in the mice previously exposed to AAV2/8-EV. The observed increase in IgG levels in the animals previously exposed to AAV2/8-EV was probably from the B cell populations that were unaffected by bortezomib treatment and reactivated in response to the administration of AAV2/8-DC190-alphaGal. Consistent with this theory was the appearance of similar levels of total IgG levels in the PBS- and bortezomib-treated animals and the fact the kinetics of the increase in total IgG was similar between the PBS- and bortezomib-treated animals (Figure 4b).

To determine whether the reduction in the levels of the pre-existing anti-AAV antibodies mediated by bortezomib affected gene transduction by AAV2/8-DC190-alphaGal, the levels of α-galactosidase A in the serum were measured. No enzyme was detected in the PBS- and bortezomib-treated mice (Figure 4b). In contrast, mice that were naïve to any treatment showed abundant expression of α-galactosidase A following the administration of AAV2/8-DC190-alphaGal (Figure 4c), and levels were consistent with previous reports [16], [17]. Immunohistochemical staining for α-galactosidase A in liver sections showed similar results (Figure 4d). Taken together, these data suggest that despite the ability of bortezomib to deplete plasma cells, the remaining memory B cells that were not affected by bortezomib were reactivated in response to the challenge with AAV2/8-DC190-alphaGal. These data also indicate that residual antibody levels and the subsequent rapid production of large amounts of anti-AAV-specific IgG blocked infection by the recombinant AAV2/8-DC190-alphaGal vector and therefore the expression of α-galactosidase A in the liver.

## Discussion

AAV-mediated gene transfer is emerging as a platform for treating a variety of disease indications. This increasing acceptance is a result of the vector’s versatility at transducing various cell types and its relatively attractive safety profile [18], [23]–[27]. However, the clinical deployment of AAV, particularly for treating diseases of the viscera, is limited by the presence of pre-existing immunity against the virus in the general population. Anti-AAV immunity consists of two major components, specifically, humoral and cellular, which limit the transduction efficiency of the virus and the expression of the transgenes. A major contributor to the inhibition of AAV-mediated transduction is the prevalence of neutralising anti-AAV antibodies in humans due to their prior exposure to the virus [6]. Cellular immune responses to the virus, mediated by antigen-specific cytotoxic CD8^+^ T cells, also eliminate virally infected cells. In this study, bortezomib, a specific inhibitor of the 26 S proteasome that reportedly has multiple effects on cellular and humoral immune responses [9], was evaluated for its ability to attenuate pre-existing immunity to AAV. Although bortezomib efficiently reduced the level of anti-AAV immunity, this effect was insufficient to facilitate subsequent transduction by an AAV vector of a similar serotype.

The 26 S proteasome is an essential component of the cytosolic protein degradation machinery, which is involved in the turnover of normally folded proteins and the degradation of misfolded proteins [28]. Misfolded proteins trigger the so-called unfolded protein response, which targets their removal by the proteasome system. Because bortezomib efficiently inhibits the proteasome, it can block unfolded protein response, thereby inducing cellular apoptosis [29]. Plasma cells are terminally differentiated B cells that secrete large amounts of IgG [20], [30], [31]. Because of this high level of protein synthesis (increased occurrence of misfolded proteins), plasma cells are hypersensitive to proteasome inhibitors, such as bortezomib. This inhibitory property of bortezomib has been demonstrated in a pre-clinical model of systemic lupus erythematosus and in a clinical setting in the treatment of multiple myeloma, which is a B cell-derived malignancy in which cancer cells produce large amounts of secreted IgG [9], [15], [29], [32]. In this study, we examined whether this property of bortezomib could be used to deplete anti-AAV antibody-producing cells. A reduction in anti-AAV-specific IgG levels might allow subsequent infection by a recombinant AAV vector.

To mimic conditions in humans, mice were first exposed to a recombinant AAV vector (AAV2/8-EV) to elicit the production of high levels of anti-AAV antibodies. Treatment of these mice with bortezomib reduced the titres of antibodies and the CD8^+^ cytotoxic T cell responses. However, these beneficial effects were insufficient to facilitate subsequent gene transduction after the systemic administration of a second recombinant AAV vector (AAV2/8-DC190-alphaGal). Following the administration of the second vector, anti-AAV antibody levels rose rapidly, presumably resulting from combination of residual antibody levels and the reactivation and subsequent differentiation of AAV-specific memory B cells. Consistent with this theory, a decrease in CD138^+^ plasma cells (but not memory B cells or other B-cell populations) was detected following treatment with bortezomib. This finding regarding the selectivity of bortezomib is congruent with reports by Neubert et al [13]. Memory B cells have been characterised as producers of IgG at lower levels; therefore, they may be less sensitive to the action of proteasome inhibitors [20]. Although we surmise that residual antibody levels after bortezomib treatment contributed significantly to the lack of ability to re-infect mice with AAV2/8-DC190-alphaGal (as relatively low levels of neutralizing anti-AAV antibodies are sufficient to block AAV infection [33]), we do not believe that this was simply due to the inability of the bortezomib treatment to reduce anti-AAV antibody titres to a level below 8,000. In bortezomib-treated animals, there was no difference in the kinetics of the increase in the anti-AAV titre in the sera of mice following infection with AAV2/8-DC190-alphaGal between the PBS- and bortezomib-treated groups (Figure 4), thereby indicating that there was an active expansion of the number of antibody-producing cells. The data shown in Figure 4a also demonstrate that anti-AAV antibody levels rose with significantly slower kinetics in previously naïve mice than in animals previously exposed to AAV2/8-EV, indicating a primary response in the previously naïve mice vs. a secondary memory response in the previously AAV2/8-EV-infected animals. These data suggest that bortezomib only affects B cells, such as plasma cells, that are actively synthesising large amounts of IgG. It is also important to point out that we observed bortezomib-related toxicity in ∼15% of animals treated with 1 mg/kg bortezomib, whereas there was no measurable effect when we used 0.2 or 0.04 mg/kg bortezomib indicating that bortezomib displays a narrow therapeutic window as a strategy to lower anti-AAV immunity in the mouse strain we used (Figure 1a and data not shown). Bortezomib also causes considerable side effects in human subjects treated with this agent, although the therapeutic window for clinical use may be very different [9], [32]. Taking this into consideration also guided the design of our studies to limit the exposure of mice to bortezomib. A dose of 1.3–1.5 mg/m^2^ body surface area is typically used to address multiple myelomas in human. This converts to approximately 0.04 mg/kg for a 60 kg person. Using a biometrics conversion factor of 10∶1 between mouse and human [34], the human dose is roughly equivalent to 0.4 mg/kg in mouse. Thus, our effective dose at 1 mg/kg in mouse approximates that used in the human setting; hence, the clinical dose in human may be sufficient to reduce pre-existing antibodies to various antigens. Yet, this dose led to toxicity in mice which may be due to species-specific differences.

The sustained rise in antibody levels in both PBS- and bortezomib-treated animals was somewhat unexpected. AAV clears from circulation of mice 2–4 hours post tail vein injection [35]. The fact that anti-AAV levels rise for weeks after administration of AAV2/8-DC190-alphaGal suggests that antigens from AAV may be retained for extended periods of time in secondary lymphoid organs to activate B cell populations and once again highlights the importance of depleting memory B cells that home to secondary lymphoid organs in the interest of efficient administration of vector.

Interestingly, although the antibody levels were similar in bortezomib- and PBS-treated mice following the administration of AAV2/8-DC190-alphaGal (Figure 4b), the number of CD138^+^ plasma cells was lower in the bortezomib-treated mice than in the PBS-treated mice (Figure 2a, left panel and Figure 2b, top row; Figure 2c, left panel and Figure 2d, top row). This finding suggests that re-activation of AAV-specific memory B cells did not cause the cells to differentiate into CD138^+^ plasma cells or that these cells were not located in the anatomical locations examined. It is also possible that the re-activated memory B cells differentiated into a developmental stage that was still CD138^−^. Additional studies are needed to differentiate between these possibilities. B cells have the capability to secrete IgG at many stages during their development [31], and, because the bortezomib treatment was discontinued at the time of re-infection, re-activated memory B cells were not inhibited from producing AAV-specific IgGs. This treatment regimen (i.e., not administering bortezomib during the second infection with AAV2/8) was chosen because the primary goal of this study was to evaluate bortezomib as a depleting agent for IgG-producing plasma cells and to examine the effects of this depletion on subsequent AAV infections. We designed our studies to achieve this goal with the shortest exposure of mice to bortezomib as possible (see above). Bortezomib treatment during the second infection would have interfered with memory T cell-mediated immune responses, which we believe would have confounded the conclusions of the studies on the AAV-specific plasma cells.

We designed our experiments to elicit strong immune responses against AAV in mice by using a relatively high amount of AAV. The dose we used has been routinely used for pre-clinical studies in our previous studies [16]–[18]. Additionally, kinetics of anti-AAV responses show similar kinetics to what we detected in our studies at significantly lower doses of AAV (e.g. 10^10^/mouse, reference [36]). As a reference, the recently published clinical studies targeting Factor IX deficiency in humans used a dose range of 2*10^11–^2*10^12^ vector genomes/kg [37]. We believe that the dose we used in our studies approximates the doses used in this human study taking into account conversion factors between mice and humans [34]. Whether bortezomib would have shown better efficacy at lower doses of initial AAV infection remains to be determined as little is known about the effect of antigen dose on B cell memory formation.

The administration of AAV2/8-DC190-alphaGal to mice that were previously exposed to AAV2/8-EV did not cause measurable expression of α-galactosidase A. This result is probably due to the neutralisation of AAV2/8-DC190-alphaGal by the anti-AAV humoral immune response. The lack of transgene expression was not due to competition for transcription factors because the AAV used in the first infection did not encode a transgene and harboured a different promoter (the CMV promoter is used in AAV2/8-EV, whereas the DC190 promoter is used in AAV2/8-DC190-alphaGal). We also do not believe that the protocol of prolonged bortezomib treatment itself interfered with re-infection of mice with AAV2/8-DC190-alphaGal as bortezomib displays a fast clearance rate (half-life in blood of bortezomib is <8 h [38], [39]) that is significantly shorter than the time period (3 days) we allowed between the final bortezomib injection and re-infection with AAV2/8-DC190-alphaGal.

Taken together, the data indicate that lowering pre-existing immune responses against AAV to a level that will enable efficient transduction by recombinant AAV vectors is likely to remain challenging. The bortezomib-mediated reduction in the levels of AAV-specific IgG was not sufficient to facilitate a repeat transduction by a recombinant AAV2/8-based vector. Because bortezomib has a narrow therapeutic window, a combination therapy that includes additional B cell inhibitors, such as anti-CD20 antibodies (e.g. Rituximab), may be required to reduce efficiently the pre-existing humoral immunity to AAV. The data presented here highlight the need to dramatically reduce the levels of AAV-specific IgG and argue for the elimination of AAV-specific memory B cells that have the capability to produce AAV-specific antibodies upon re-activation. Further studies are needed to evaluate the efficacy of a combination treatment that targets both plasma and memory B-cell populations.
